# Clinical evaluation of real-time artificial intelligence provision of expert representation in indocyanine green fluorescence angiography during colorectal resections

**DOI:** 10.1097/JS9.0000000000002136

**Published:** 2024-11-15

**Authors:** Ashokkumar Singaravelu, Philip D. Mc Entee, Niall P. Hardy, Mohammad F. Khan, Jurgen Mulsow, Conor Shields, Ronan A. Cahill

**Affiliations:** a UCD Centre for Precision Surgery, University College Dublin; b Department of Surgery, Mater Misericordiae University Hospital; c Department of Surgery, Mater Private Hospital, Dublin, Ireland

## Introduction

Highlights

- Artificial intelligence models that digitally represent in real-time how expert surgeons interpret indocyanine green fluorescence angiography (ICGFA) signalling for transection-level selection during colorectal resection have been developed and tested live in the operating theatre.
- Deep learning algorithm demonstrated generalisability among ICGFA users from other institution, and it’s learning from multiple expert surgeons can represent a shared expert view regarding transection level perfusion.
- This automated method to help new users gain confidence in ICGFA interpretation could help the results now being achieved in clinical trials disseminate into wider use.

Indocyanine green fluorescence angiography (ICGFA) use in colorectal resectional surgery, especially regarding intestinal level for transection^1^, is associated with significantly reduced postoperative anastomotic leak (AL) rates^2,3^. Whilst ICGFA can be consistently interpreted with experience, human factors (including learning curve and distraction), tissue (wherein ICG also moves by diffusion), and camera (especially illumination disparity across the field of view) behaviours mean grey areas in interpretations can exist^4–7^. Here, we develop and evaluate artificial intelligence (AI) methods that represent in real-time during surgery how experienced ICGFA users interpret visualised ICGFA colorectal signalling.

## Methods

Computational explorations and software development were performed via a prospective registered study (NCT 04220242, Institutional Review Board approval reference 1/378/2092) and are reported here via DECIDE-AI^8^.

### Patients

Consenting patients undergoing elective colorectal resection without postoperative AL provided intraoperative ICGFA and clinical data for model training-testing. ICGFA recordings visualised (PINPOINT Endoscopic Fluorescence System, Stryker Corp), either intracorporeally or extracorporeally, the colonic segment intended for transection after meso-colic preparation for at least 90 s after intravenous ICG (0.1 mg/kg, Verdye, Diagnostic Green) including indication of actual transection site initially by one surgeon (RC, 15 years ICGFA experience) and then including interpretations of two others (MFK, 10 years; NPH, 6 years). Additional anonymised ICGFA recordings (*n*=10, one with AL) from other surgeons (*n*=7), including other imagers (*n*=4, Stryker 1688 and Arthrex SynergyID) were also tested against their own and our surgeons’ interpretations along with ileal segments visualised in right hemicolectomy recordings. Three other test videos demonstrated ICGFA-realised poor perfusion (addressed intraoperatively).

### Algorithm development

ICGFA frames underwent stabilisation via affine geometric transformation with warping ensuring feature alignment with the initial frame in synchronous white light and near-infrared (NIR) imagery (MATLAB R2024a). Four fluorescence intensity-time related features (maximum intensity, upslope, and times to maximum and 50%-maximum) were computed from segmented (72×96 grid) NIR frames (360×480 pixels). Four lines of differing lengths and positions across the fluorescence-nonfluorescent boundary in each intestinal segment were sampled from training-validation videos for data augmentation (35-pixel high rectangular regions centred on each line). A median value was calculated at every co-ordinate and the data condensed back into a single linear representation. The site of stapling by the operating surgeon was labelled ‘expert’ for training with the perfused area proximal labelled ‘good’ and the contralateral nonperfused area labelled ‘poor’. For non-PINPOINT test imagery, curve features were extracted from overlay displays by formula (frame green channel–RGB) and converted to grayscale before processing.

1. Point-based approach (machine learning, ML): classifies each point on an intestinal line using 10-fold cross-validation for training with weighted K-Nearest Neighbour modelling.
2. Sequence approach (deep learning, DL): To classify points as a linear sequence, two bidirectional long short-term memory models were trained as five-layered (sequence input, bi-LSTM (200 hidden units), fully connected, soft-max and classification output) network. The early stop technique avoided overfitting.

### Evaluation metrics

Predictions were overlaid on white light imagery with green (‘Expert’), red (‘Good’), and blue (‘Poor’) denotation. Pixel-level metrics evaluated categorisation of ‘expert’ points on the line. Object-level accuracy was calculated as number of times the actual stapler placement fell within the boundaries of the DL-predicted ‘expert’ zone.

### Implementation

Trained algorithms were tested on unseen videos including prospectively in consecutive patients in theatre (18/01/2024-09/05/2024) in real-time via laptop (11th Gen Intel(R) Core i7-1185G7, NVIDIA-T500 2GB), double-blinded to the surgeons.

See supplementary for interpretability of AI methods (Supplemental Digital Content 1, http://links.lww.com/JS9/D536).

## Results

Training involved 20 patient videos (80 bowel regions, total/average area 506 625/6255 pixels^2^) and validation five (20 regions, 131 775/6588 pixel^2^) (Table S1, Supplemental Digital Content 1, http://links.lww.com/JS9/D536) for ML and DL initially with, training taking 1.8 and 5 s and overall validation pixel-level accuracies being 67.8% and 70.3%, respectively. However, ML did not create a zone distinguishing the ‘expert’ stapler site separate from ‘good’ and so only DL advanced to object-level testing, including a second DL algorithm (3DL) trained with additional ICGFA expert annotations (240 regions, total/average area 1 545 075/6437 pixel^2^, with five video validation from 60 regions, total/average area 408 100/6802 pixel^2^ trained in 18 s with 67.7% validation pixel-level accuracy). Such testing involved 28 unseen videos (34 bowel segments) (Table 1).

**Table 1:** Pixel-level metrics for predicted ‘expert’ label in unseen test cases, both prospective in theatre testing (with the same ICGFA expert on whom the method was trained) and in post-hoc testing (against other experienced ICGFA users from other institutions including other imaging systems).

	Accuracy	Precision	Recall	Specificity	F1-score	DICE
Overall unseen testing						
ML	0.64	0.32	0.31	0.76	0.29	0.29
DL	0.85	0.73	0.98	0.80	0.81	0.81
3DL	0.88	0.75	0.93	0.85	0.81	0.81
Prospective in-theatre testing (*n*=15)						
ML	0.63	0.33	0.32	0.75	0.29	0.29
DL	0.89	0.77	0.98	0.86	0.85	0.85
Additional post-hoc testing (*n*=10)						
ML	0.64	0.29	0.27	0.77	0.27	0.27
DL	0.73	0.58	0.91	0.67	0.67	0.68
3DL	0.88	0.75	0.93	0.85	0.81	0.81

A DICE index of 1 means perfect overlap (size of the stapler=size of predicted ‘expert’ zone) (note: 1 stapler diameter=~12 grid modules). High recall indicates that the model is effective in identifying ‘expert’ points on the line (i.e. predicted ‘expert’ points are within the actual staple placement site).

3DL, Deep learning model trained on three experts; DL, Deep learning method trained on one expert; ML, Machine learning method.

**Figure 1:**
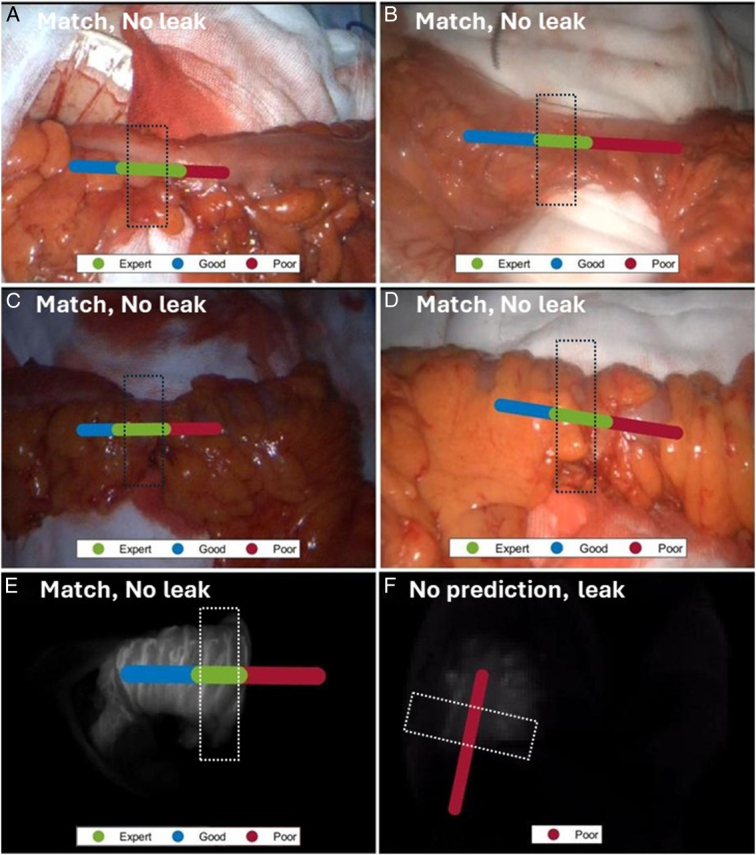
Examples of prediction overlays of DL algorithm, tested both prospectively in theatre (A–D) and post-hoc (E,F), demonstrating the correspondence between the actual stapler placement (dotted box) and DL ‘expert’ zone predictions alongside patient anastomotic leak outcomes (i.e. leak/no leak).

Prediction overlays from 30 s of ICG inflow were provided on average 13 s with 100% accurate at object-level including in three poor perfusion cases (where only red zoning displayed) (Fig. 1). In video recordings by other surgeons (four live in theatre), DL matched the perfusion interpretation of its trainer in 10/11 (90.9%) and that of other surgeons in 9/11 (81.8%). In the discordant cases, no prediction was made in one who suffered AL while the prediction was five grids proximal to the actual stapler site in the other.

## Discussion

Our ‘surgical eye’ develops experientially through witnessing others’ performances. Intraoperatively, even senior surgeons consult others to view the operative scene. Here, an AI method, grounded in expert ICGFA interpretations in nonleak cases, takes the ‘surgical peer’ role and represents digitally how dynamic ICGFA imagery would be interpreted by an experienced user. Previous work has detailed that experienced users’ interpretation of ICGFA correlates highly, can be relayed by intensity-time series curve milestones and can be applicable to ML methods^5,9^.

Of the two models, the bi-LSTM model performed more usefully, likely because its prediction generation includes sequencing across well and poorly-perfused bowel segments, while the ML model was unable to predict a stapling zone. Neither model learned off raw video frames; instead validated curve parameters from stablised video footage are extracted^10^. DL algorithms demonstrated generalisability among ICGFA users from other institutions, indicating it’s learning from multiple experts can represent a shared expert view, and correctly predicted poor perfusion and, in one case, AL.

Despite the relatively limited applications, this AI method demonstrates encouraging discrimination that can be rapidly scaled using advanced hardware–software interface technology albeit in a relatively small number of patients, NIR systems and centres. Advanced trialling, including more mal-perfusion and AL cases and additional expert interpreters, may provide better confidence. Although not fully explainable, DL activation visualisations (supplementary) are supportive of the method.

Therefore, if ICGFA becomes the standard of care, the DL method demonstrated here could usefully provide a ‘helping hand’ with low cognitive load for new and uncommon ICGFA adopters and so will be further advanced including useability in broader observational blinded study. The method also has application in documentation of correct ICGFA use and interpretation.

## Ethical approval

Computational explorations and software development were performed via prospective registered study (NCT 04220242, Institutional Review Board approval reference 1/378/2092).

## Consent

Ethics committee approval and informed consent were obtained.

## Source of funding

This study was supported in part by a Disruptive Technologies Innovation Fund Award by The Government of Ireland via Enterprise Ireland. The study sponsor had no involvement in the collection, analysis and interpretation of data and in the writing of the manuscript, and in the decision to submit the manuscript for publication.

## Author contribution

R.C. and A.S.: conceptualization, methodology, and writing – original draft; A.S. and P.M.E.: data curation; A.S.: formal analysis, software, and visualisation; R.C.: funding acquisition, project administration, resources, and supervision; A.S. and P.M.E.: investigation; N.P.H., M.F.K., J.M., and C.S.: validation; R.C. and P.M.E.: writing – review and editing.

## Conflicts of interest disclosure

The work disclosed here is the subject of a patent filed in the area by University College Dublin. Professor Ronan A Cahill also receives speaker fees from Stryker Corp and Ethicon/J&J, consultancy fees from Arthrex, Astellas, Diagnostic Green and Touch Surgery (Medtronic), research funding from Intuitive Corp and Medtronic as well as, recently, from the Irish Government (DTIF) in collaboration with IBM Research in Ireland and from EU Horizon 2020 in collaboration with Palliare and, currently, from Horizon Europe in collaboration with Arctur. Other authors report no industry disclosures.

## Research registration unique identifying number (UIN)

Computational explorations and software development were performed via prospective registered study (NCT 04220242, Institutional Review Board approval reference 1/378/2092).

## Guarantor

Professor Ronan A. Cahill, Consultant General and Colorectal Surgeon, Mater Misericordiae University Hospital, Dublin, Ireland. E-mail: ronan.cahill@ucd.ie and Ashokkumar Singaravelu.

## Data availability statement

Available on provision of registered protocol.

## Provenance and peer review

Not commissioned, externally peer-reviewed.

## Supplementary Material

SUPPLEMENTARY MATERIAL
