# Supplementary material for: Clinical evaluation of real-time artificial intelligence provision of expert representation in indocyanine green fluorescence angiography during colorectal resections
Source: Int J Surg. 2024 Nov 15;110(12):8246–9. doi: 10.1097/JS9.0000000000002136 (PMC11634173; doi:10.1097/JS9.0000000000002136)
Supplement: SUPPLEMENTARY MATERIAL [file js9-110-8246-s001.docx]

**Clinical evaluation of real-time artificial intelligence representation of expert interpretation in indocyanine green fluorescence angiography for colorectal resections**

Ashokkumar Singaravelu^1^, Philip D Mc Entee^1,2^, Niall P Hardy^2^, Mohammad Faraz Khan^2^, Jurgen Mulsow^2^, Conor Shields^2^, Ronan A. Cahill^1,2^

^1^UCD Centre for Precision Surgery, University College Dublin, Ireland

^2^Department of Surgery, Mater Misericordiae University Hospital, Dublin, Ireland

^3^Department of Surgery, Mater Private Hospital, Dublin, Ireland

**Corresponding author.**

Prof Ronan A. Cahill,

47 Eccles Street, Dublin 7, Ireland.

Email: [ronan.cahill@ucd.ie](mailto:ronan.cahill@ucd.ie)

Telephone: 00353 1 716 4597

ORCID ID: 0000-0002-1270-4000

Twitter: @MaterSurgery

**Supplementary Materials - Index**

| **Supplementary Methods** |  |
| --- | --- |
| Algorithm Development – Sequence Approach | *pag. 2* |
| Interpretability techniques | *pag. 2* |
| **Supplementary Results** |  |
| Interpretability | *pag. 2* |
| **Supplementary Figures and Tables** |  |
| Table S1 | *pag. 3* |
| Figure S1 | *pag. 4* |
| Figure S2 | *pag. 4* |

**Supplementary Methods**

## **Sequence approach (deep learning, DL)**

Each line was considered as a sequence and a bidirectional long short-term memory (bi-LSTM) model was trained to make predictions for each point on the line. The model was trained using the adaptive moment estimation (Adam) algorithm as the solver. The bi-LSTM network comprised 200 hidden units, 60 maximum number of epochs, and gradient threshold of 2. Validation patience was employed to stop the training early to avoid overfitting. State and gate activation functions are *tanh* and *sigmoid* respectively. Input weights was 1600 x 4 single (initialiser ‘*glorot’*). Recurrent weights was 1600 x 200 single (initialiser ‘*orthogonal’*). Bias was 1600 x 1 single (initialiser ‘*unit-forget-gate*’). Hidden state and cell state were 400 x 1 single. Input, recurrent and bias weights learn rate factor were 1. Loss function was *cross entropy ex*.

## **Interpretability techniques**

Partial dependence plots were used to illustrate the relationship between predictor and classification prediction scores. These were generated to explain how the weighted k-nearest neighbour model makes predictions for the entire data set. Visualization of activations of the bi-directional long short term memory (bi-LSTM) model, a type of interpretability technique, was employed to explain network predictions. A heatmap was created by extracting the activations of first 100 hidden units of the bi-LSTM layer corresponding to a test sequence, where higher values indicate stronger activation.

**Supplementary Results**

Twenty-five patient videos were used for training and validation, and algorithms were tested prospectively in theatre on eighteen patients (see Table T1 for patient demographics and operative details). Please see the video demonstrating the application of deep learning algorithms in theatre in three cases (three anterior resections) with Pinpoint imaging system, and in two post hoc cases with Arthrex SynergyID^TM^ on a sigmoid resection, and with SPY-PHI (Stryker) on a right hemicolectomy.

## **Interpretability**

Partial dependence plots are shown in Figure S1. The plotted lines represent the relationship between the feature variable value on the x axis and the predicted score for each output labels on the y axis. For example, regions with lower maximum fluorescence intensity are more likely to be predicted as “poor” compared to “expert” or “good”. The activations heatmap of test sequence 1 is shown in Figure S2. The activations were extracted from each hidden unit and shows how strongly each unit activates.

**Supplementary Figures and Tables**

**Table S1.** Patient demographics and operative details.

|  | **Training**  **(n = 20)** | **Validation**  **(n = 5)** | **Prospective testing**  **(n = 20)** |
| --- | --- | --- | --- |
| **Leak** | 0 | 0 | 0 |
| **Age (years)*** | 69 (62.5-73) | 49 (47-51) | 67 (55-71.5) |
| **Male: Female** | 14:6 | 4:1 | 11:8 |
| **Cancer: Benign** | 13:7 | 5:0 | 11:8 |
| **Neoadjuvant therapy** | 3 (15%) | 1 (20%) | 3 (15.8%) |
| **Operative type** | | | |
| **Anterior resection** | 9 | 2 | 6 |
| **Sigmoid colectomy** | 6 | 1 | 2 |
| **Right hemicolectomy/**  **Ileocolic resection** | 5 | 2 | 7 |
| **Completion colectomy** | 0 | 0 | 1 |
| **Left hemicolectomy** | 0 | 0 | 1 |
| **Hartmann’s procedure/**  **Abdominoperineal resection** | 0 | 0 | 3 |

* Values are median (IQR). Two recordings were performed on one patient during prospective testing, including both the initial transection site and the revised transection site.


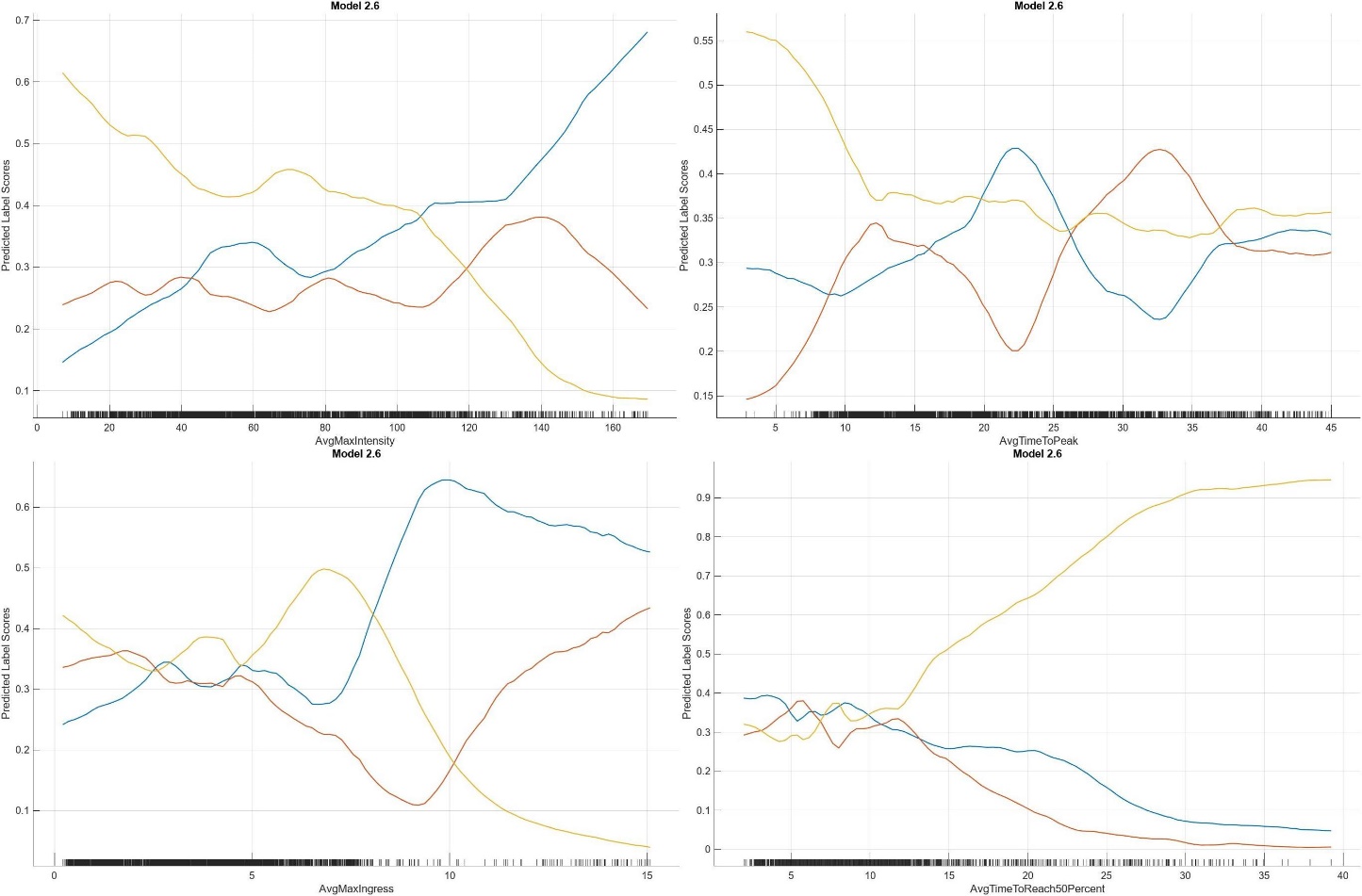


**Figure S1.** Partial dependence plots of the weighted KNN model for maximum fluorescence intensity (top left), time to peak (top right), upslope (bottom left) and time to reach 50% of maximum intensity (bottom right). Yellow = Poor, Red = Good, Blue = Expert.


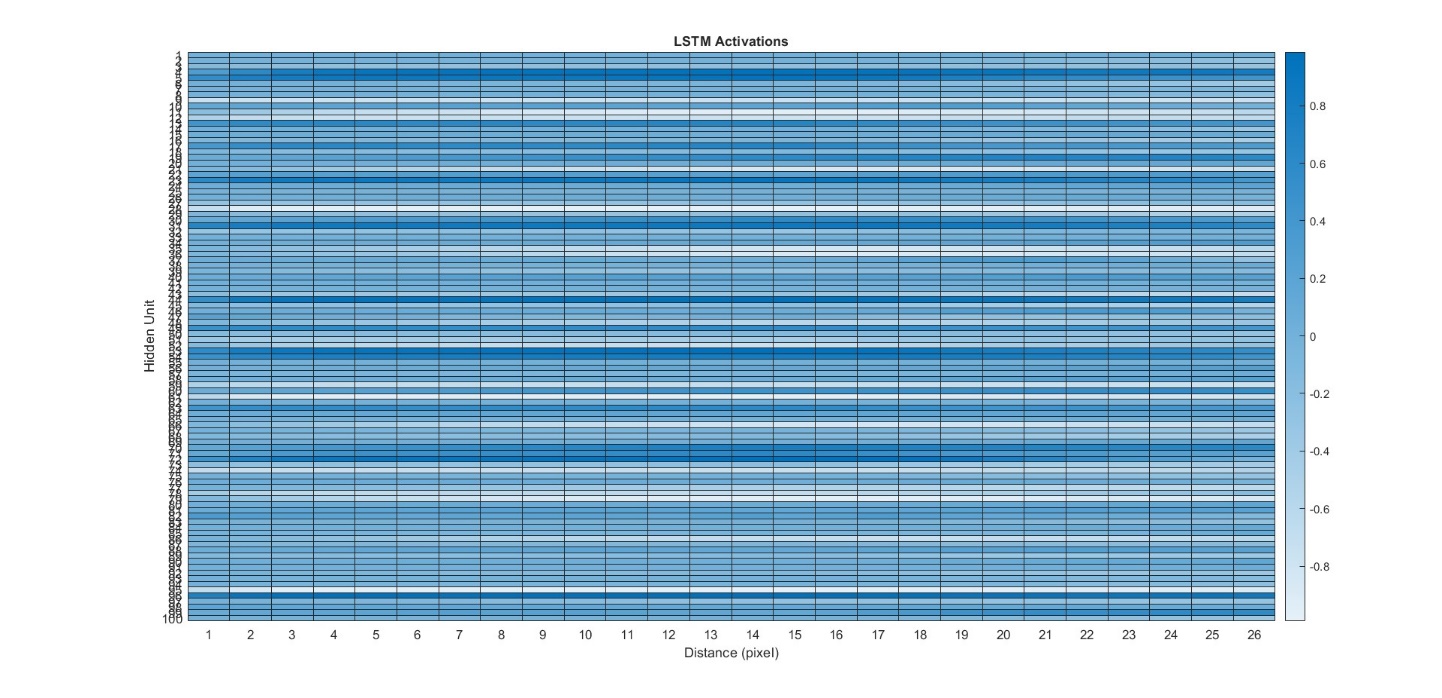


**Figure S2.** Heatmap of activations of the LSTM model for test sequence 1.
